# Climatic, socioeconomic, and migratory factors on the epidemiological dynamics of cutaneous leishmaniasis in Colombia, 2007–2021

**DOI:** 10.1371/journal.pntd.0013594

**Published:** 2025-10-13

**Authors:** Johanna Tapias Rivera, Ruth Martínez-Vega, Wendy Lorena Quintero-García, Dayana Sofía Torres-Martínez, Angela Liliana Monroy-Díaz, Lusayda Sánchez-Corrales, Juan David Gutiérrez-Torres

**Affiliations:** 1 Facultad de Ciencias Exactas, Naturales y Agropecuarias, Universidad de Santander, Bucaramanga, Santander, Colombia; 2 Escuela de Medicina, Instituto Masira, Facultad de Ciencias Médicas y de la Salud, Universidad de Santander, Bucaramanga, Santander, Colombia; 3 Posgrados en Enfermedades Infecciosas, Instituto Masira, Facultad de Ciencias Médicas y de la Salud, Universidad de Santander, Bucaramanga, Santander, Colombia; 4 Facultad de Ciencias de la Salud, Universidad de Boyacá, Tunja, Boyacá, Colombia; 5 Facultad de Ciencias de la Salud, Universidad Católica de Manizales, Manizales, Caldas, Colombia; 6 Instituto Masira, Facultad de Ciencias Médicas y de la Salud, Universidad de Santander, Bucaramanga, Santander, Colombia; London School of Hygiene and Tropical Medicine, UNITED KINGDOM OF GREAT BRITAIN AND NORTHERN IRELAND

## Abstract

The World Health Organization considers cutaneous leishmaniasis (CL) to be one of the most important neglected tropical diseases (NTDs). The application of geostatistical models, in conjunction with sociodemographic and environmental factors, enables the understanding of disease epidemiology and facilitates the implementation of targeted measures and effective case management. This research aimed to evaluate the association between climatic, sociodemographic, and socioeconomic factors with the monthly CL incidence rate at the municipality level in Colombia from 2007 to 2021. An ecological study was conducted, including laboratory-confirmed notifications of CL reported in municipalities located below 1,700 meters above sea level through the National Public Health Surveillance System. Climate data were sourced from NASA, and sociodemographic and socioeconomic variables were obtained from the National Planning Department. Hierarchical spatio-temporal regression models within a Bayesian framework were used to analyze the monthly CL. A total of 121,828 cases of CL were analyzed, with an annual median of 7,605 cases. Standardized incidence rates (SIR) ranged from 0 to 16,072 per 100,000 population (median: 105.7; IQR: 46.7-419). Eight of the 11 studied factors were associated with the monthly cases of CL: rainfall, urban dimension, and Venezuelan migration were associated with a decrease in CL cases, while qualitative housing deficit, internal migration, the multidimensional poverty index, the index of unmet basic needs, and forest coverage were associated with an increase in CL cases. CL incidence in Colombia fluctuated during the study period, with high spatial heterogeneity linked to climatic, sociodemographic. and socioeconomic factors. These findings highlight the necessity for customized territorial approaches to the prevention and control of CL, emphasizing the importance of considering municipal characteristics and aligning strategies with the Colombian Strategic Plan for CL.

## Author summary

Cutaneous leishmaniasis (CL) remains one of the most important tropical diseases worldwide, according to the World Health Organization. By using mathematical models, researchers can better understand how this disease behaves and identify the most effective ways to control it. Our study looked at over 120,000 CL cases reported across Colombia between 2007 and 2021, examining how factors like weather patterns, poverty levels, migration, and environmental conditions influenced the disease’s spread. The data were collected from NASA and Colombian government sources, and showed that most cases occurred in young adult men. Out of the 11 factors we studied, 8 were associated with the frequency of CL cases. Interestingly, we found that heavy rainfall, urban environments, and immigration from Venezuela were associated with fewer CL cases. On the other hand, poverty, internal migration within Colombia, and forested areas were associated with more cases. These results tell us that prevention efforts need to be adapted to each region’s specific conditions, taking into account local environmental and social factors that affect the disease’s spread.

## Introduction

Cutaneous leishmaniasis (CL) is a disease caused by more than 20 species of the protozoan parasite *Leishmania* and transmitted to humans through the bite of the female sandfly (Diptera: Psychodidae) of the genus *Lutzomyia* [1]*.* The increase in anthropogenic activities in forested areas and environmental disturbances are important drivers of the number of CL cases, as these factors may contribute to the adaptation and expansion of phlebotomine sandflies populations into human-modified environments, including urban and peri-urban areas [2].

CL is an endemic public health problem in nearly 100 countries, with 350 million people at risk and nearly two million new cases each year [3]. Given its varied clinical presentation, it implies a wide range of differential diagnoses and potential underreporting [4]. Furthermore, CL is considered by the World Health Organization (WHO) as one of the most important neglected tropical diseases (NTDs) because of its close relationship with socioeconomic conditions, malnutrition, population mobility, and climate [5]. CL and other clinical forms of leishmaniasis have been reported to contribute to increases in mortality and disability-adjusted life years (DALYs) (127.64%; 95% UI 95.33–180.25) from 1990 to 2019 [6].

The transmission of CL results from a complex interaction between the parasite, its vectors, and hosts, all of which are modulated by environmental and economic factors, such as urbanization, rural population, mining activities, human development, sanitation, and access to basic public services, as well as sociodemographic factors, including migration and armed conflicts, which have been associated with CL cases [7–10]. These variables interact dynamically, promoting the persistence and expansion of the disease in specific contexts. From an environmental perspective, climate plays a central role in the ecology of the vector. In this regard, factors such as temperature, relative humidity, and rainfall determine the geographical distribution of phlebotomine sand flies and influence the viability of the parasite within the vector [11,12]. For instance, an increase in ambient temperature can accelerate the development of *Leishmania* in the vector’s gut, allowing the sand fly to become infective in a shorter time [13]. In addition, changes in precipitation patterns can alter vegetation and vector microhabitats, favoring vector proliferation even in areas previously considered non-endemic. In fact, the WHO has identified climate change as one of the emerging factors that could facilitate the spread of leishmaniasis into higher latitudes and altitudes where the vector was previously absent [14].

Economically, CL disproportionately affects impoverished communities where populations often live in precarious housing conditions, and have limited access to healthcare services [15]. This delays timely diagnosis and treatment, increasing both morbidity and disease transmission [16]. Poverty may also limit the implementation of preventive measures such as the use of insecticide-treated bed nets or improvements in environmental infrastructure [17].

On a sociodemographic level, rapid and unplanned urbanization has been identified as a key driver in the reemergence of disease foci. Urban expansion into peri-urban or rural areas, often accompanied by deforestation, alters the natural habitats of vectors and reservoirs, creating new opportunities for both zoonotic and anthroponotic transmission [18]. Outbreaks of leishmaniasis are frequently associated with abrupt changes in human environments, including social conflicts, mass migration, and the breakdown of vector control programs [19,20]. Together, these factors create an epidemiological landscape in which leishmaniasis cannot be fully understood through a purely biomedical lens, but rather requires a comprehensive approach that considers the ecological, social, and economic dimensions of the affected regions [19]. Moreover, CL transmission is increasing in Latin America; among the 10 countries with the highest number of cases worldwide, three are in the Americas (Brazil, Colombia, and Peru), together accounting for up to 75% of the estimated global incidence [18,19]. The incidence of CL cases in Colombia increased from 9.9 cases per 100,000 inhabitants in 2007 to 34.2 in 2016, with the Amazon region having the highest incidence, ranging from 76 to 240 cases per 100,000 inhabitants [21]. In Colombia, CL is caused by multiple zoonotic *Leishmania* species, including *Leishmania (Viannia) braziliensis*, *L. (V.) guyanensis*, and *L. (V.) panamensis* [22]*;* the latter being the most frequently reported. The transmission cycle is predominantly zoonotic, with wild mammals such as rodents (*Melanomys caliginosus*), sloths (*Choloepus hoffmanni*), and marsupials (*Didelphis* spp.) serving as reservoir hosts [23]. These reservoirs play a critical role in maintaining the parasite in sylvatic environments and enabling spillover to human populations in peri-urban areas, especially where interventions such as agriculture, deforestation, livestock rearing, and land-use change increase human contact with natural habitats [24,25].

Many vector-borne diseases exhibit a distinct seasonal pattern, suggesting their sensitivity to climate, precipitation, temperature, relative humidity, and other climatic variables that affect both vectors and the pathogens they transmit in different ways [24]. In this regard, the El Niño-Southern Oscillation (ENSO), characterized by anomalous temperatures in the equatorial Pacific Ocean, can change atmospheric and climatic patterns, leading to high temperatures, decreased precipitation, alteration of river flow rates, and low atmospheric humidity in the Andean, Caribbean, and Pacific regions. This phenomenon has been associated with the potential distribution of vector species and CL cases in some regions of Colombia [26]. Also, ecological niche models have identified variations in the potential distribution of reservoirs such as *Didelphis marsupialis, Bradypus variegatus*, and wild rodents according to the ENSO phase [7,8,26,27].

The application of geostatistical models, in association with socioeconomic, sociodemographic, and environmental factors, has enabled the mapping of CL, serving as a useful tool for understanding the epidemiological behavior of diseases and facilitating the implementation of measures focused on vector control and case management [9,10].This research aimed to evaluate the association between temperature and rainfall, as well as sociodemographic and socioeconomic factors, and the monthly CL rate at the municipality level in Colombia from 2007 to 2021, taking into account spatio-temporal autocorrelation.

## Methods

### Study design and population

An ecological study was conducted. According to the National Administrative Department of Statistics (DANE), Colombia had 51,049,498 inhabitants in 2021 living in 1,122 local administrative entities, of which 986 (87.9%) are located below the minimum municipal altitude of 1,700 meters above sea level (masl), the minimum altitude threshold for CL in the country. This cutoff was selected to avoid the inclusion of allochthonous cases in the analysis.

### Data cases

All laboratory-confirmed cases of CL were obtained from the National Public Health Surveillance System, reported from 2007 to 2021. In Colombia, a laboratory-confirmed case of cutaneous leishmaniasis is defined as a patient from an endemic area presenting with cutaneous lesions and at least three of the following criteria: absence of trauma history, evolution longer than two weeks, ulcers, nodular lesions, satellite lesions, or localized lymphadenopathy, with confirmation of Leishmania parasites by parasitological, histopathological, or molecular methods [28]. Daily cases were aggregated by month for each municipality based on the reported onset date of the symptoms provided by each patient. Cases with inconsistency in the municipality of occurrence or date were excluded from the study. Cases lacking information on gender or age were excluded from the analysis. Additionally, the islands of San Andrés and Providencia were omitted due to the unavailability of climate data.

### Climate and environmental data

Monthly municipality rainfall and temperature data from 2007 to 2021 were downloaded from National Aeronautics and Space Administration (NASA) product GLDAS_NOAH025_M version 2.1 with a spatial resolution of 0.25 degrees [29]. Municipality annual forest cover and deforestation percentages were obtained from the NASA product MCD12Q1 Version 6.1 (500-meter resolution) [30] and Hansen 2013, global_forest_change_2022_v1_10, with a resolution of 30 meters [31], respectively. The R package raster version 3.4 [32] was used to perform the data extraction between the raster files for rainfall, temperature, forest cover, and deforestation with the municipal polygons, and estimate the average value for each municipality.

### Socioeconomic data

The socioeconomic data were obtained from the TerriData repository of the National Planning Department [33], as well as the assessment of municipal typology and territorial breaches used for the socioeconomic characterization of the municipalities [34]. The socioeconomic indicators by municipality included urban dimension, rural Gross Domestic Product (GDP), qualitative housing deficit, internal migration, Venezuelan migration, barriers to health access, the Multidimensional Poverty Index (MPI), and the Index of Unmet Basic Needs (UBN) (S1 Table). These data are only available for the year 2018, because they were measured during the last national census. This unique value was included based on the consideration that, in Colombia, changes in the socio-economic conditions of a municipality typically take several decades. Consequently, different observations of the same socio-economic variables tend to remain consistent throughout the study period.

### Statistical analysis

The dataset was compiled by integrating data from multiple sources, using the DANE code to identify each municipality. CL cases were described by sex and age groups (0–19 years, 20–39 years, 40–59 years, 60–79 years, and 80 and over). Age- and gender-standardized incidence rates (SIR) by municipality for the period were calculated using the direct method, based on the average municipal population and the average Colombian population from DANE between 2007 and 2021. In addition, the SIR and factors were described using the median, interquartile range, and minimum and maximum values. The epidemiological curve and the SIR map were constructed in Stata 16.0.

### Spatiotemporal model

Hierarchical spatio-temporal regression models within a Bayesian framework were developed to estimate the association of CL cases with the climate, environmental, and socioeconomic factors, considering the spatial distribution of the Colombian municipalities and variations across the 180-month study period, with a zero-inflated Poisson (ZIP) distribution, described as:


$$PrPr\;(Y=\text{0})\;=\;\pi +(\text{1}-\;\pi )e^{{-\lambda }_{it}}$$ (1)

$$PrPr\;\left(Y=y_{it}\right)\;=\;(\text{1}-\;\pi )\frac{\lambda^{y_{it}}\;e^{{-\lambda }_{it}}}{y_{it}!}$$ (2)

where *π* is the probability of extra zeros, *y*_*it*_ is the standardized count of CL cases, and *λ*_*it*_ is the population for the *i*^*th*^ municipality in the *t*^*th*^ month, respectively.

The linear predictor was defined on the logarithmic scale as:


$$log\left(y_{it}\right)\;=\;\alpha +\;\lambda_{it}+\;\beta_{x}X_{xi}+\;s_{i}+\;u_{i}+T_{t}$$ (3)

where α is the intercept, *X* represents the vector of covariates (climate, environmental, and socioeconomic factors) with their respective regression coefficients *β*_*x*_, the parameters *s* and *u* represent the spatially structured and the unstructured residuals (i.e., spatial component) according to the Besag-York-Mollie specification [35], and *T* represents the effect of the temporal component. The spatial structure was defined via an adjacency matrix with a queen specification (i.e., including all neighboring municipalities that share a border with each municipality). In this context, municipalities are considered neighbors if they share either a common boundary or a single point (i.e., corner). This “queen” criterion provides a more inclusive neighborhood definition than the “rook” specification, which only considers shared edges. The queen-based adjacency matrix is particularly suitable for geographic units with irregular shapes or small areas, where corner adjacency may represent meaningful spatial interaction. The temporal component *T*_*t*_ was set using a non-parametric formulation that incorporates both structured and unstructured effects, similar to the spatial component.

The predictor model with a non-parametric specification and a spacetime interaction was defined as:


$$log\;log\;\left(y_{it}\right)\;=\;\alpha +\;\lambda_{it}+\;\beta_{x}X_{xi}+\;s_{i}+\;u_{i}+\gamma_{t}+\;\varphi_{t}+\;\partial_{it}$$ (4)

Where ∂_*it*_ is the interaction between the unstructured spatial and temporal effects (*u*_*i*_ and φ_*t*_) specified with a Gaussian exchangeable prior ∂_*it*_ ∼ Normal $\left(\text{0},\;\frac{\text{1}}{\tau_{\partial }}\right)$ [22].

The conditional precision of the temporally structured and space-time interaction effects was assigned with logGamma (0.1, 0.01) prior. To assess potential collinearity among the independent variables, Pearson’s correlation coefficients were calculated. A correlation was considered substantial when the coefficient was greater than 0.6. Additionally, a univariate analysis was performed to evaluate the crude association between each variable of interest and the outcome. Variables with significant or theoretically relevant associations were considered for inclusion in the multivariable models. The best-fit model was identified using the deviance information criterion (DIC), and the 95% credible interval (95% CI) was estimated.

The statistical analysis was performed with the R-INLA package version 23.04.24. The data and the scripts in R and STATA to reproduce the results are available at: https://github.com/juandavidgutier/leishmaniasis_spatial_temporal.

## Results

### Cases of cutaneous Leishmaniasis

**Fig 1:**
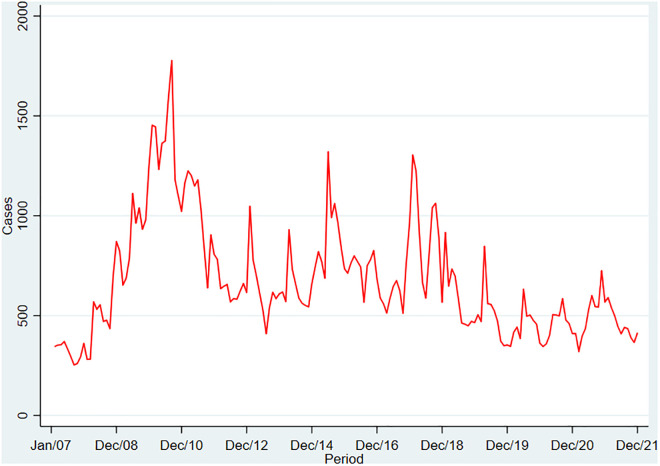
Reported cases of Cutaneous Leishmaniasis in Colombia from 2007 to 2021.

A total of 984 Colombian municipalities located below 1,700 masl were analyzed, documenting 121,828 new cases of CL reported between 2007 and 2021. These cases were reported from 940 (95.5%) municipalities. The median number of cases per year was 7,605 (IQR:5,714–10,267). The year 2009 recorded the highest number of total cases (14,736), while 2007 reported the lowest (3,779). Fig 1 illustrates the monthly frequency of CL cases during the period. Notably, there was a considerable increase from 2007 to 2009, reaching the highest peak in January 2010 (1,777 cases). Subsequently, the second-highest monthly frequency occurred in January 2014 (1,320 cases), followed by March 2016 (1,304 cases). Afterward, a gradual decrease in monthly cases was observed. January was the month with the highest number of cases each year.

**Fig 2:**
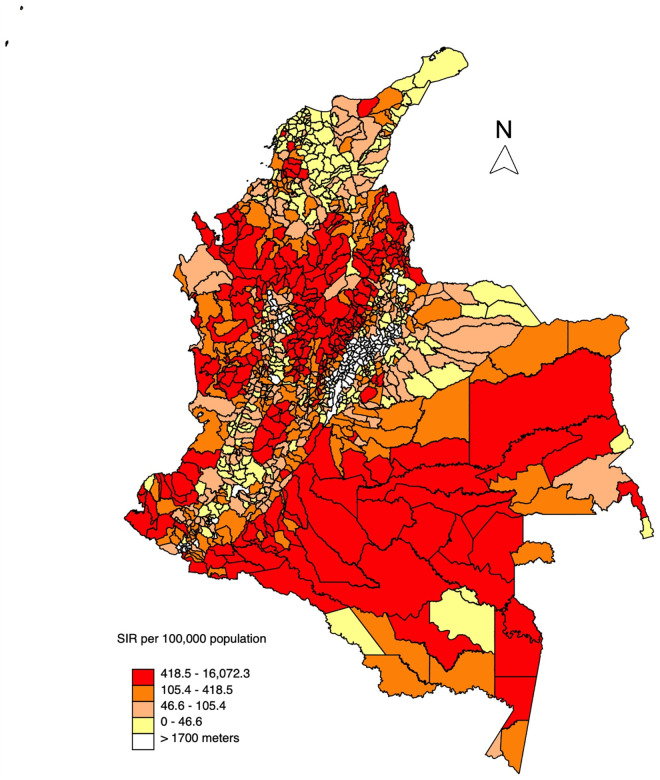
Standardized incidence rate of CL in Colombian municipalities located below 1,700 masl. Map created and modified by the authors using STATA version 16 and spatial data from the Instituto Geográfico Agustín Codazzi (IGAC), available at Colombia en Mapas (https://www.colombiaenmapas.gov.co/) Municipal Boundaries – Colombia en Mapas/ IGAC. (https://www.colombiaenmapas.gov.co under a CC BY 4.0 license [36].

Most cases were observed in males (79.2%, 96,534 cases). People aged 20–39 accounted for 53.6% of cases (68,590 cases), followed by those aged 0–19 (27.2%, 33,193 cases) and 40–59 (11.9%, 14,521 cases). The SIR during the study period at the municipal level ranged from 0 to 16,072 cases per 100,000 population (median: 105.7; IQR: 46.7–419). In 383 (38.9%) municipalities, the SIR exceeded 418.5 cases per 100,000 population (Fig 2). La Macarena, in Meta, had the highest SIR during the period, followed by Valdivia, in Antioquia, and Santa Helena del Opón, in Santander, which together accounted for 5.3% of the cases (S2 Table). Notably, three of the top ten CL SIR municipalities are located in Santander (S2 Table).

### Descriptive analysis of climatic, environmental, and socioeconomic factors

Average monthly temperature and rainfall fluctuated considerably during the period. The median monthly temperature was 20.9°C (IQR: 16.8 - 25.2) (Table 1). Precipitation varied widely, ranging from 0.13 mm to 706.2 mm. Forest cover had a median of 11.6%, with values ranging from 0.01% to a maximum of 100%, while deforestation ranged from 0 to 5%. The urban dimension index had a median of 0.06 (IQR: 0.04 - 0.09), indicating a predominance of rural characteristics across municipalities, as at least 75% of them scored below 0.10 (Table 1).

**Table 1:** Description of the climatic, environmental, and socioeconomic factors.

Factor	Measurement frequency	Municipalities included	n	Median	P25	P75	Min	Max
**Temperature (°C)**	Monthly	984	177,120	20.90	16.78	25.20	4.17	32.75
**Rainfall (mm)**	Monthly	984	177,120	203.93	131.34	278.30	0.13	706.16
**Forest cover (%)**	Yearly	984	14,760	11.57	1.09	39.13	0.01	100
**Deforestation (%)**	Yearly	984	14,760	0.12	0.05	0.29	0	5.04
**Urban dimension (index 0–1)**	Once	984	984	0.06	0.04	0.09	0.001	1
**Qualitative housing deficit (%)**	Once	964	964	43.58	30.29	57.82	1.60	89.16
**Internal migration (%)**	Once	964	964	11.84	8.07	16.25	2.12	37.02
**Venezuelan migration (%)**	Once	960	960	0.58	0.17	1.80	0	32.37
**Barriers to health access (%)**	Once	984	984	4.35	2.40	6.80	0	40.20
**MPI (%)**	Once	984	984	42.10	32.00	54.30	4.50	98.50
**UBN index (%)**	Once	984	984	18.91	12.06	30.98	1.59	95.96

n, number of observations; p25, percentile 25; p75, percentile 75; UBN, unmet basic needs; MPI, multidimensional poverty index; Min, minimum; Max, maximum.

The qualitative housing deficit varied widely, with a minimum of 1.6 and a maximum of 89.2, highlighting the need for improved housing infrastructure in most municipalities. The median internal migration rate was 11.8%, while Venezuelan migration was 0.58% (Table 1). It is important to note that data on the qualitative housing deficit and internal migration were not available for 20 municipalities, while data on Venezuelan migration were not available for 24 municipalities (S3 Table).

Although the median value for barriers to health access was low (4.4%), the highest value (40.2%) indicates inequalities in healthcare access among municipalities, which could delay the diagnosis and treatment of leishmaniasis, exacerbating its impact on public health. Socioeconomic conditions influence the susceptibility of populations to CL. MPI had a median of 42.1%, while the UBN index was 18.9%, indicating that a large portion of the population lives in poverty. This increases their vulnerability to vector-borne diseases due to limited access to adequate infrastructure and healthcare services.

### Climatic, environmental, and socioeconomic factors associated with monthly CL cases

In the univariate analysis, eight of the 11 studied factors were associated with the monthly cases of CL. Rainfall, urban dimension, and Venezuelan migration were associated with a decrease in the cases, while qualitative housing deficit, internal migration, MPI, UBN index, and forest coverage were associated with an increase in the cases. Temperature, barriers to healthcare access, and deforestation were not associated with CL cases. Given that the MPI and the UBN index were highly correlated, two multiple models were constructed, each including one of these variables. The associations observed in the univariate analysis were maintained in the multivariable analysis. However, model 1, which included MPI, had a better fit, as indicated by a lower DIC (Table 2).

**Table 2:** Factors associated with the monthly standardized incidence rate of cutaneous leishmaniasis in Colombian municipalities with a minimum altitude below 1,700 mals.

Factor	Crude		Model 1		Model 2	
	SIRR	95% CI	SIRR	95% CI	SIRR	95% CI
**Temperature**	1.006	0.965 -1.050	NI	NI	NI	NI
**Rainfall**	0.966	0.960 - 0.975	0.966	0.957 - 0.976	0.965	0.954 - 0.975
**Urban dimension**	0.540	0.514 - 0.566	0.647	0.612 - 0.684	0.618	0.587 - 0.650
**Qualitative housing deficit**	1.479	1.381 - 1.583	1.152	1.086 - 1.122	1.196	1.131 -1.265
**Internal migration**	1.164	1.080 - 1.254	1.188	1.121 - 1.259	1.156	1.093 - 1.222
**Venezuelan migration**	0.691	0.643 - 0.741	0.861	0.812 - 0.913	0.851	0.801 - 0.900
**Barriers to health access**	1.044	0.983 - 1.108	NI	NI	NI	NI
**MPI**	1.759	1.640 - 1.887	1.235	1.150 - 1.326	NI	NI
**UBN index**	1.326	1.224 - 1.436	NI	NI	1.085	1.019 - 1.155
**Forest cover**	1.185	1.130 - 1.243	1.135	1.085 - 1.187	1.162	1.112 - 1.212
**Deforestation**	0.994	0.986 - 1.003	NI	NI	NI	NI
**DIC**			659068.1		693949.7	

SIRR, age-standardized incidence rate ratio; UBN, unmet basic needs; MPI, multidimensional poverty index; DIC, deviance information criteria; CI, confidence intervale; NI, not included.

## Discussion

The epidemiological landscape of CL in Colombia reflects the interaction of social, environmental, and demographic factors. From analysis of 121,828 cases across 940 municipalities we observed that CL incidence was positively associated with qualitative housing deficit, internal migration, MPI, UBN index, and forest coverage, and negatively associated with precipitation, urban dimension, and Venezuelan migration.

CL remains a significant public health concern in Colombia due to its extensive geographical distribution and the vulnerability of a substantial portion of the population. Although the number of cases has declined in recent years, the data reveal a fluctuating trend, likely influenced by changes in social determinants of health and environmental factors [37].

The Pan American Health Organization, has indicated that CL transmission follows a predominantly sylvatic pattern, linked to forest-related work activities as reflected. in the higher prevalence among men aged 20–50 years [37]. These findings align with our results, which show that 53.6% of cases occurred among individuals aged 20–39 years. However, the 0–19 age group also presents a significant number of cases, which could suggest the presence of a peridomestic transmission pattern, highlighting the importance of implementing strict surveillance [38]. Also, in the Colombian context, the displacement of populations from rural areas to marginalized urban settlements exacerbates the risk of CL, as these environments often lack basic infrastructure and create favorable conditions for vector presence [37].

Our findings demonstrated that munipalities with higher qualitative housing deficit, MPI, and UBN index, reported CL cases, align with previous studies across Latin American and suggesting a broader regional pattern [19,39]. Conversely, municipalities with higher urban development had lower CL incidence, suggesting that urban development may reduce exposure by improving housing quality, sanitation, and access to preventive measures. This finding emphasizes that tackling CL requires not only biomedical interventions but also policies to reduce poverty and improve living condition.

Migration emerged as another relevant factor. Internal migration was positively associated with the incidence of CL cases, in line with evidence showing that population mobility, driven by conflict, economic pressures, or the search for job opportunities, can increase exposure to vectors [7,40,41]. Additionally, historical armed conflicts in Colombia have altered population dynamics and CL epidemiology, frequently exposing indigenous populations and military personnel, to vector contact as they pass through various geographical areas [10]. This has led to the collapse of healthcare services, mass displacement of people, and the spread of the disease to new areas, resulting in outbreaks in neighboring countries that have received refugees [9].

Environmental dynamics also represent a particularly complex aspect of this research. The positive correlation with forest coverage and CL incidence highlights the complex ecological interactions that govern vector transmission. This is consistent with environmental studies in the Amazon Basin, which have linked landscape transformations with vector proliferations and disease incidence [34]. At the same time,

rainfall was negatively associated with CL regression models This relationship may be attributed to the susceptibility of vectors to climatic factors, such as temperature and precipitation [42]. Similar findings have been reported in municipalities of Colombia, including Caquetá, Antioquia, and Tolima with tropical environmental characteristics, where negative correlations between CL incidence and precipitation were observed [43].

Likewise, in other Latin American regions, CL cases have been associated with ENSO events, which involve marked changes in rainfall patterns [44], affecting vector niche and reservoir host populations.. These shifts can contribute to changes in parasite maintenance in sylvatic cycles and increase spillover risk to humans, particularly in deforested or agricultural frontiers. Furthermore, it should be noted that the relationship between temperature, precipitation, and CL cases is not linear, considering the multifactorial nature of this relationship, including human behavior [42]. While our study observed negative association with rainfall, other clinical forms of leishmaniasis, such as visceral leishmaniasis, have shown a positive relationship with rainfall and increased abundance of immature vector forms in Colombia [19,45].

The socioeconomic and environmental interplay also highlights the broader context of CL as a neglected tropical disease. Colombia is a country with extensive tropical regions and a poverty rate of around 13%, faces compounded challenges fromclimate change, which is projected to increased drought frequency and poverty rates. These conditions may create a synergistic that amplifies CL transmission [46]. Evidence from other Latin American regions has shown that agricultural activity, rural residence inadequate sanitation, and limited access to essential public services further elevate the risk to CL [47–49]. However, in Colombia, there is not always a linear relationship between CL cases and agricultural conditions underscoring the importance of considering multiple overlapping determinants [50].

The socioeconomic determinants emerging from the analysis provide critical insights into disease vulnerability. The strong correlation between the MPI, internal migration, and CL incidence reveals a stark geographical and social gradient of disease burden. These findings resonate with global research on NTDs, which emphasizes the profound link between social marginalization and infectious disease transmission [51].

The limitations of the study are mainly related to data quality, including possible underreporting of CL cases in national databases. Also, some cases had to be excluded (n = 29) due to missing information on age, gender, symptoms onset date, sex, and municipality. In addition, since climate data were not available for the islands of San Andrés and Providencia, these municipalities were excluded. However, only one case was reported in these islands. An additional limitation of this study is that the monthly climatic data were aligned with the reported date of symptom onset, which may not precisely correspond to the actual time of transmission due to the incubation period. This temporal misalignment could introduce uncertainty in the estimation of associations between environmental variables and incidence. Lastly, the socioeconomic data was only available once during the study period (for 2018). Furthermore, the national surveillance system does not report species-level identification, preventing species-specific analyses that could provide more nuanced insights into the transmission dynamics.

Finally, one important limitation of our study is the assumption of linear relationships between covariates and CL incidence, which may oversimplify the complex ecological and epidemiological dynamics of transmission. Environmental variables such as temperature and rainfall likely exhibit non-linear associations with disease transmission, as extreme values can be detrimental to sandfly survival and parasite development, while socioeconomic variables may demonstrate threshold effects where improvements in living conditions show diminishing returns beyond certain levels. Future studies should explore non-linear relationships using flexible modeling approaches such as splines or higher-order random wlak models within the INLA framework, to better capture and inform targeted public health interventions.

Despite these limitations, this study reconceptualizes CL from a purely biomedical condition to a complex socio-ecological phenomenon rooted in interconnected environmental, economic, and demographic systems. The spatial analysis of these multidimensional relationships provides novel insights into transmission dynamics in resource-limited settings. The spatio-temporal autocorrelated model moves beyond traditional static epidemiological frameworks, instead characterizing CL as a dynamic process intrinsically linked to its geographic and temporal context.

## Conclusions

In this study, the strong association between the incidence of CL and socioeconomic factors such as a qualitative housing deficit, internal migration, the UBNI and MPI underscores the role of structural vulnerabilities in maintaining transmission. Furthermore, the positive association between forest cover and the incidence of CL highlights the prevalence of the sylvatic zoonotic transmission cycle in Colombia, particularly in municipalities below 1700 masl. Collectively, these findings emphasize the importance of implementing territorially differentiated and intersectional strategies for CL prevention and control. These strategies must be adapted to the specific socio-environmental contexts of each municipality and aligned with national priorities, such as Colombia’s Strategic Plan for CL. Additionally, integrating spatial and environmental data into public health planning could enhance early warning systems and the targeting of vector control programs, improve access to healthcare and resource allocation, and facilitate a comprehensive approach to the social determinants of health to reduce the burden of CL and achieve sustainable disease control.

## Supporting information

S1 Table Definitions and descriptions of variables included in the study. (DOCX)

S2 Table Top ten municipalities ranked by standardized incidence rate of cutaneous leishmaniasis, 2007–2021. (DOCX)

S3 Table Municipalities with no available data on qualitative housing deficit, internal migration, or external migration. (DOCX)
