# Supplementary material for: Climatic, socioeconomic, and migratory factors on the epidemiological dynamics of cutaneous leishmaniasis in Colombia, 2007–2021
Source: PLoS Negl Trop Dis. 2025 Oct 13;19(10):e0013594. doi: 10.1371/journal.pntd.0013594 (PMC12517516; doi:10.1371/journal.pntd.0013594)
Supplement: S1 Table — (DOCX) [file pntd.0013594.s001.docx]

**S1 Table.** **Definitions and descriptions of variables included in the study**

| **Variable** | **Description** |
| --- | --- |
| **Urban dimension (index 0–1)** | Index reflecting the degree of urbanization in a municipality, based on population density, service coverage, and urban concentration. Normalized from 0 (highly rural) to 1 (highly urban). |
| **Qualitative housing deficit (%)** | Percentage of dwellings with structural deficiencies, insufficient space (including mitigable overcrowding), or lacking basic public services. |
| **Internal migration (%)** | Percentage of residents who migrated from another department or municipality within Colombia. Reflects internal mobility patterns. |
| **Venezuelan migration (%)** | Percentage of residents born in Venezuela. based on data from the 2018 National Census and territorial typology. |
| **Barriers to health access (%)** | Percentage of individuals reporting difficulties accessing healthcare services due to institutional, informational, or documentation-related barriers. |
| **Multidimensional Poverty Index (MPI, %)** | Percentage of households identified as multidimensionally poor, based on deprivations in education, health, housing, and basic services. |
| **Unmet Basic Needs Index (UBN, %)** | Percentage of households with at least one critical unmet basic need, including inadequate housing, lack of access to services, poor school attendance, or severe overcrowding. |
