# Supplementary material for: Climatic, socioeconomic, and migratory factors on the epidemiological dynamics of cutaneous leishmaniasis in Colombia, 2007–2021
Source: PLoS Negl Trop Dis. 2025 Oct 13;19(10):e0013594. doi: 10.1371/journal.pntd.0013594 (PMC12517516; doi:10.1371/journal.pntd.0013594)
Supplement: S2 Table — (DOCX) [file pntd.0013594.s002.docx]

**S2 Table. Top ten municipalities ranked by standardized incidence rate of cutaneous leishmaniasis, 2007–2021**

| **Municipality** | **State** | **Average period SIR** |
| --- | --- | --- |
| **Nilo** | Cundinamarca | 41.12 |
| **Pueblo Rico** | Putumayo | 38.72 |
| **Santa Helena Del Opón** | Santander | 37.75 |
| **Rio Blanco** | Tolima | 36.73 |
| **Valdivia** | Antioquia | 36.12 |
| **Rovira** | Tolima | 29.23 |
| **El Carmen De Chucurí** | Santander | 28.28 |
| **San José Del Palmar** | Chocó | 27.50 |
| **Landázuri** | Santander | 25.98 |
| **San Benito** | Santander | 25.31 |
