# Supplementary material for: Climatic, socioeconomic, and migratory factors on the epidemiological dynamics of cutaneous leishmaniasis in Colombia, 2007–2021
Source: PLoS Negl Trop Dis. 2025 Oct 13;19(10):e0013594. doi: 10.1371/journal.pntd.0013594 (PMC12517516; doi:10.1371/journal.pntd.0013594)
Supplement: S3 Table — (DOCX) [file pntd.0013594.s003.docx]

**S3 Table. Municipalities with no available data on qualitative housing deficit, internal migration, or external migration.**

| **Venezuelan Migration** | **Internal Migration** | **Quality Housing** |
| --- | --- | --- |
| **Code.DANE** | **Code.DANE** | **Code.DANE** |
| 15550 | 91263 | 91263 |
| 15778 | 91405 | 91405 |
| 27413 | 91407 | 91407 |
| 27745 | 91430 | 91430 |
| 91263 | 91460 | 91460 |
| 91405 | 91530 | 91530 |
| 91407 | 91536 | 91536 |
| 91430 | 91669 | 91669 |
| 91460 | 91798 | 91798 |
| 91530 | 94343 | 94343 |
| 91536 | 94663 | 94663 |
| 91669 | 94883 | 94883 |
| 91798 | 94884 | 94884 |
| 94343 | 94885 | 94885 |
| 94663 | 94886 | 94886 |
| 94883 | 94887 | 94887 |
| 94884 | 94888 | 94888 |
| 94885 | 97511 | 97511 |
| 94886 | 97777 | 97777 |
| 94887 | 97889 | 97889 |
| 94888 |  |  |
| 97511 |  |  |
| 97777 |  |  |
| 97889 |  |  |
